# Stereopsis in animals: evolution, function and mechanisms

**DOI:** 10.1242/jeb.143883

**Published:** 2017-07-15

**Authors:** Vivek Nityananda, Jenny C. A. Read

**Affiliations:** 1 Wissenschaftskolleg zu Berlin, Institute for Advanced Study, Wallotstraße 19, Berlin 14193, Germany; 2 Newcastle University, Institute of Neuroscience, Henry Wellcome Building, Framlington Place, Newcastle Upon Tyne NE2 4HH, UK

**Keywords:** 3D, Camouflage breaking, Coarse and fine stereopsis, Range-finding

## Abstract

Stereopsis is the computation of depth information from views acquired simultaneously from different points in space. For many years, stereopsis was thought to be confined to primates and other mammals with front-facing eyes. However, stereopsis has now been demonstrated in many other animals, including lateral-eyed prey mammals, birds, amphibians and invertebrates. The diversity of animals known to have stereo vision allows us to begin to investigate ideas about its evolution and the underlying selective pressures in different animals. It also further prompts the question of whether all animals have evolved essentially the same algorithms to implement stereopsis. If so, this must be the best way to do stereo vision, and should be implemented by engineers in machine stereopsis. Conversely, if animals have evolved a range of stereo algorithms in response to different pressures, that could inspire novel forms of machine stereopsis appropriate for distinct environments, tasks or constraints. As a first step towards addressing these ideas, we here review our current knowledge of stereo vision in animals, with a view towards outlining common principles about the evolution, function and mechanisms of stereo vision across the animal kingdom. We conclude by outlining avenues for future work, including research into possible new mechanisms of stereo vision, with implications for machine vision and the role of stereopsis in the evolution of camouflage.

## Abstract (precis)

**Summary:** Stereopsis has evolved independently in different animals. We review the various functions it serves and the variety of mechanisms that could underlie stereopsis in different species.

## Introduction

**Fig. 1.**
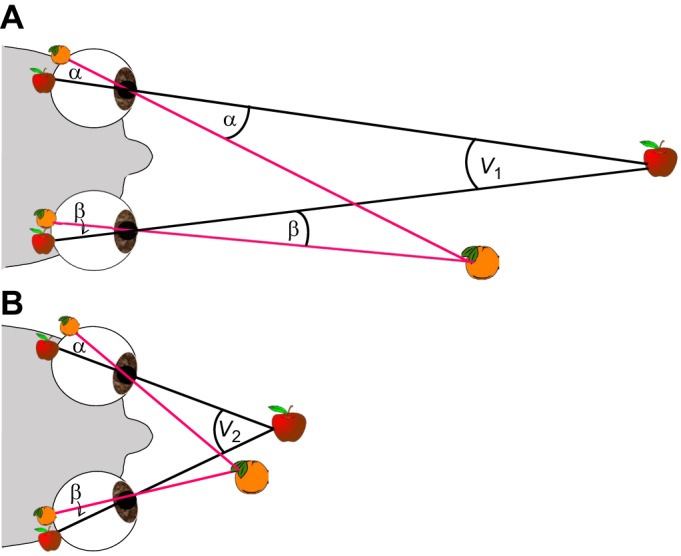
**Stereopsis in mobile eyes.** In both A and B, the apple is imaged at the fovea while the orange is to the left of the fovea in both eyes, by an angle α in the left eye and β in the right. The retinal disparity is therefore the same in both cases: the absolute disparity of the apple is 0, and the absolute disparity of the orange is α−β, which is also the relative disparity between the two objects. However, the different positions of the eyes (less converged in A, strongly converged in B) means that the locations of the objects in space is very different in the two cases. In both cases, the fact that the orange is closer can be deduced from the relative disparity, but to deduce the absolute distance to either object requires a knowledge of the vergence angle (*V*_1_, *V*_2_). This can, in principle, be derived either from extra-retinal information, such as signals from the eye muscles, or from the geometry of how objects across the visual scene project into the two eyes (Hartley and Zisserman, 2004). For animals whose eyes are fixed in the head, the same points in the two retinae always correspond to the same point in head-centred space.

Humans view the world from two front-facing eyes located approximately 6 cm apart. This offset location means that the two eyes see slightly different views of the world. For example, in Fig. 1, the person is fixating an apple, whose images therefore fall at the same location – the fovea – of both eyes. The nearer object, an orange, projects to the two retinae at slightly different distances from the fovea. This difference in retinal location is known as ‘binocular disparity’ (see Glossary). In 1838, Wheatstone demonstrated that these subtle disparities between retinal images are detected by the brain and provide a potent cue to the depth structure of the world around us (Wheatstone, 1838). This ability has come to be known as (binocular) stereopsis (see Glossary), or stereoscopic or stereo vision. Informally, it is also often called 3D vision.

In humans, stereopsis has become an attractive model system for understanding the link between neural activity and perception (Roe et al., 2007; Read, 2015a). We now have a good basic understanding of the different processes of primate stereopsis and the brain areas involved (Cumming and DeAngelis, 2001). Yet we know little about stereopsis in other species. Remarkably, stereopsis was not demonstrated behaviourally in any non-human species until 130 years after Wheatstone, with Bough's (1970) proof of stereopsis in macaque monkeys. We now know that many different species have evolved some form of stereoscopic vision. However, with the exception of a few model taxa, including macaques, cats and barn owls, we know very little about the abilities, function or neural basis of animal stereopsis.

This information is important for two quite different reasons. First, it is a prerequisite for understanding the evolution of stereopsis. As we shall see, although the basic idea behind stereopsis is straightforward, many different forms of stereopsis are possible, which make different demands on the animal and provide different types of information. We have to understand how stereopsis works in a given species before we can understand either the selective advantages it provides or the adaptations that other species may have evolved in response. Second, a less anthropocentric understanding of stereopsis could provide unexpected benefits in machine vision. Most current machine stereo algorithms are inspired to some extent by human stereopsis, which is powerful but also complex and costly. Other, more limited forms of stereopsis might be more appropriate in particular situations.

This Review aims to bring together recent developments in animal, human and machine stereopsis and show how a better understanding of stereopsis across the animal kingdom could provide fresh insights in diverse fields including ecology, evolution and engineering. We first consider the visual cues to depth and the distinctive benefits of stereopsis as compared with other ways of computing 3D structure. After discussing classic hypotheses about why stereopsis evolved, we review our current knowledge about which animals have stereopsis and the different techniques used to demonstrate this. We discuss the particular selective advantages which stereopsis may provide in different species, and consider different forms of stereopsis and how these could be implemented computationally. Finally, we outline possible future research avenues. These include investigation of new mechanisms of stereo vision in various animals, the contributions these could make to machine vision and the role of stereopsis in the evolution of camouflage.

## Depth perception and stereopsis

All sighted animals face the problem of how to derive information about a 3D world from 2D retinal images. 2D images contain a range of depth cues which can, in principle, be used to derive information about 3D structure. Depth cues can usefully be grouped into three classes (Banks et al., 2016): light transport (e.g. shading), perspective (e.g. looming; see Glossary) and triangulation (e.g. stereopsis; Fig. 1). Triangulation depth cues are based on comparing views of an object from multiple locations. This is a particularly reliable means of depth perception because it depends only on geometry, rather than on assumptions about the specific scene. Other cues require assumptions, e.g. about lighting (for shade cues) or object shape (for perspective), which can lead to incorrect perceptions when these assumptions are not met (Gregory, 1968). Indeed, light transport and perspective are jointly known as pictorial cues, because human painters exploit them to produce the illusion of depth on a flat canvas. Evolution has also discovered ways of fooling sensory systems that rely on pictorial cues, for example, counter-shading or forced perspective to mislead estimates of 3D shape and size (Cuthill et al., 2016; Endler et al., 2010; Rowland et al., 2008).

Glossary **Accommodation**

The ability of an eye to change optical power in order to keep objects at different distances in sharp focus.

**Correspondence problem**

The need to work out which points in the two eyes' images represent the same point in space. In Fig. 1, the corresponding points in the two eyes are those that both view the orange, or both view the apple. In a complex scene (e.g. Fig. 3) it can be challenging to work out which points correspond.

**Disparity**

Binocular disparity is the difference in the images of a single object as seen by the two eyes. In this paper, we define disparity as the difference in angular position on the retina, i.e. the angle α−β in Fig. 1.

**Fixation point**

The point in space viewed by both foveae (the location of the apple in Fig. 1).

**Focal plane**

For a simple eye, the set of locations in space where objects are imaged sharply on the retina.

**Looming**

The increase in the retinal size of an approaching object.

**Simple eye**

Like a human eye or a typical camera, where light is collected across a relatively large aperture and focused by a lens so as to form a sharp image on the photoreceptors.

**Stereogram**

A pair of images, one for the left eye and one for the right, constructed with disparities to create an illusion of depth. An example is shown in Fig. 3.

**Stereoscope**

An optical device for displaying stereograms using mirrors, prisms and/or lenses.

**Stereopsis**

We define this as the ability to gain information about the 3D structure of visual scenes by comparing information collected separately and simultaneously from different lines of sight to the same region of space. Other definitions exist; e.g. a few authors use the term to mean the perception of depth/solidity, however obtained.

**Vergence**

The vergence angle is the angle between the visual axes from the two eyes. The vergence angle needed to fixate an object depends only on the interocular separation and the distance to the object, meaning that vergence is a potential depth cue for mobile-eyed animals. In animals whose eyes move in the head, convergence refers to turning the eyes inwards to view near objects, while divergence refers to turning them back outwards to view more distant objects (cf. Fig. 1A and B).

Perhaps the most basic triangulation cue is that provided by views collected at different times by an eye translating relative to an object or scene. When the eye is static, we refer to this as ‘structure from motion’. When the eye is moving, we refer to this as ‘motion parallax’. The term ‘optic flow’ is used to refer to the pattern of motion across large regions of the visual field in either situation. This type of information is extremely powerful, and as far as we know all sighted animals use it to some extent. Its great disadvantage is that it requires motion. If the visual scene is static, an animal can access this information only by moving its eye in space, either while flying as bees do (Kirchner and Srinivasan, 1989) or with movements such as the bobbing head movements of birds (Frost, 1978) and the side-to-side peering head movements of insects (Poteser and Kral, 1995; Wallace, 1959). These movements risk giving away the animal's position to either prey or predators.

Less obviously, in simple eyes (see Glossary), light rays passing through different regions of the pupil also offer triangulation-type depth cues (Banks et al., 2016). Simple eyes are efficient because they collect light across a wide pupil, and use a lens to focus light coming from different directions onto different retinal locations, thus forming an image. However, this only works for objects at one particular distance, the focal plane (see Glossary). Objects nearer or further than the focal plane suffer defocus blur. Humans and some other animals, such as squid and chameleons, are able to use this depth cue (Chung and Marshall, 2014; Harkness, 1977; Ott et al., 1998). Monochromatic defocus blur is ambiguous about the sign of depth (whether the object is nearer or further than the focal plane) (Held et al., 2012). However, signed information is potentially available from higher-order optical aberrations. Defocus blur also varies with the wavelength of light, and this chromatic aberration could also provide a signed depth cue to species with colour vision. Humans exploit depth information from higher-order and chromatic aberrations (Fincham, 1951), but currently nothing is known about whether other simple-eyed species use these cues. A related type of triangulation cue is focal accommodation (see Glossary). If a simple eye has the ability to vary its optical power, information about object distance is potentially available from the power required to bring the object into focus on the retina. Animals such as owls (Wagner and Schaeffel, 1991), toads (Douglas et al., 1986; Jordam et al., 1980) and chameleons (Harkness, 1977; Ott et al., 1998) use accommodation cues to help them judge distance.

The triangulation-class cue that is the focus of this Review is, of course, stereopsis. We adopt a slightly unusual definition of stereopsis as ‘the ability to gain information about the 3D structure of visual scenes by comparing information collected separately and simultaneously from different lines of sight to the same region of space’. Note that our definition excludes blur, because blur pools information from different lines of sight, and excludes motion parallax because that uses information acquired non-simultaneously. Unlike most definitions of stereopsis, we do not specify that stereopsis should be binocular. Certainly, the different lines of sight in stereopsis usually are acquired by the two eyes (Fig. 1). But the compound eye of stomatopods collects light from intersecting lines of sight, potentially allowing triangulation within a single eye (Schiff et al., 1985). If this were proven, we would regard it as a form of stereopsis. However, because monocular stereopsis is currently only theoretical, in the rest of this Review we shall discuss binocular stereopsis.

## Binocular vision and the evolution of stereopsis

Two views dominated the early discussion of the evolution of stereo vision. These have been classified as the special and general hypotheses (Fox, 1978). The former argued that stereo vision evolved in mammals and is most advanced in primates. The latter argued that ‘stereopsis comes along with [binocular vision] as a sort of psychological windfall’ (Walls, 1942), and so would be present in any animal with a substantial region of space viewed by both eyes. Several lines of evidence initially seemed to support the special theory of stereo vision evolution, including anatomical specializations in the visual systems of humans and monkeys (e.g. frontal eyes, semi-decussation of the optic tract; Fox, 1978) and the discovery of disparity-sensitive neurons in different mammals (Clarke et al., 1976; Ptito et al., 1991). Over subsequent years, several independent studies demonstrated stereo vision in multiple animals, including non-mammalian ones (Clarke et al., 1976; Collett, 1977; Fox et al., 1977; Nityananda et al., 2016a; Ptito et al., 1991; Rossel, 1983; Timney and Keil, 1999; van der Willigen et al., 1998), giving more support to the general hypothesis.

However, while it is clear now that stereo vision has evolved multiple times in different evolutionary lineages, this does not necessarily mean that every organism with a binocular overlap is capable of stereopsis. Binocular vision is costly, requiring additional photoreceptors and/or a reduced field of view and/or reduced acuity. But it offers advantages other than stereopsis: redundancy (the critical frontal region is still visible even if one eye is blinded; Jones and Lee, 1981), improved signal-to-noise ratio under poor lighting conditions, and the ability to see around occluders in a cluttered environment (Blake and Wilson, 2011; Harris and Wilcox, 2009; Changizi and Shimojo, 2008). These advantages could lead binocular vision to be selected for, even without the additional extraction of depth information. Although animals with a binocular overlap face the challenge of fusing the two images into a single view of the world, most neuroscientists no longer subscribe to Wall's (1963) view that stereopsis comes free as a ‘psychological windfall’ along with binocular single vision. The existence of stereoblind humans, who show no stereopsis despite good acuity in both eyes and binocular single vision (Richards, 1970), is evidence against that. In primates at least, stereopsis requires a costly neuronal architecture spanning several cortical areas (Cumming and DeAngelis, 2001; Welchman, 2016). Thus, even in animals that have a binocular overlap, further careful work is needed to demonstrate stereo vision.

## Demonstrating stereopsis

As we have seen, many depth cues are potentially available, so it is surprisingly hard to demonstrate conclusively that an animal is using stereopsis. Preliminary behavioural experiments can compare animals' assessments of depth-based stimuli when viewed monocularly compared with binocularly. Such experiments, for example, showed that horses (Timney and Keil, 1999) and mantises (Maldonado and Rodriguez, 1972) made inaccurate depth judgements when viewing stimuli with only one eye. For a conclusive demonstration, however, we need a method of manipulating the disparity between the eyes, without affecting monocular information. One approach is to place prisms in front of the eyes so as to shift the images in opposite directions in each retina, altering binocular disparity without affecting the image's position averaged across both eyes. Prisms have been used in this way to demonstrate stereopsis behaviourally in toads and in praying mantises (Collett, 1977; Rossel, 1983). Toads fitted with prisms that manipulated disparity cues made more errors in estimating the distance of prey (Collett, 1977). In a similar experiment, Rossel (1983) placed prisms in front of the eyes of mantises and presented them with an approaching fly. Mantises in these experiments reached out to capture the fly based on disparity rather than non-triangulation-based depth cues. Prisms were also used in early neurophysiological experiments on sheep; these experiments indicated that the sheep cortex contains neurons tuned to binocular disparity (Clarke et al., 1976).

More general methods that allow arbitrary stimuli to be presented to each eye were developed first for humans. The oldest are the Wheatstone and Brewster stereoscopes (see Glossary), in which arrangements of mirrors, prisms and/or lenses direct different images to each eye (Brewster, 1856; Wheatstone, 1838). These have been widely used in primate behavioural experiments, where responses are typically given via eye movements, and in cat and primate neurophysiological experiments (e.g. Cumming and Parker, 1997, 1999).

More recently, optical filters have been developed where the left and right eye images are displayed on a single screen but are separated by spectral content, optical polarisation or time (Baker et al., 2016; Pastoor and Wöpking, 1997). These are much more convenient than stereoscopes using mirrors or prisms, but allow a certain amount of interocular ‘crosstalk’, where an image intended for one eye is partially visible to the other. Filters are particularly useful for behavioural experiments in which the animal is required to move. Most behavioural demonstrations of stereopsis have relied on training animals to differentiate between stimuli with different stereo content. This approach has been successfully used to demonstrate stereopsis in horses, owls, falcons and macaques (Fox et al., 1977; Poggio et al., 1985; Timney and Keil, 1999; van der Willigen, 2011; van der Willigen et al., 1998). In all these cases, animals were fitted with spectral or polarisation-based filters by which different views could be shown to each eye. Subsequently, the animals learnt to differentiate flat stimuli from stimuli where depth was conveyed using stereo cues, and to further distinguish between stimuli where these cues conveyed differing (non-flat) depths.

Spectral filters have also been used to investigate stereopsis in the praying mantis (Nityananda et al., 2016a,b), exploiting natural behaviour without the need for training. Mantises spontaneously strike at prey-like virtual stimuli when these are presented with a disparity indicating that they are within the animal's catch range. Stereopsis may also be present in two other insects – dragonflies (Olberg et al., 2005) and robber flies (Wardill et al., 2017) – but conclusive tests have not yet been performed.

There is thus a substantial body of literature demonstrating stereo vision in non-primate and non-mammalian systems. This has led to the conclusion that stereopsis has evolved independently at least four times: in mammals, birds, amphibians and insects (Pettigrew, 1986). The ‘special’ hypothesis for the evolution of stereo vision is therefore disproven. Stereopsis cannot have been inherited from the common ancestor of these taxa, because binocular vision evolved independently in mammals, birds and amphibians (Pettigrew, 1986), and eyes themselves evolved independently in insects. It remains unclear whether stereopsis has evolved in all animals with binocular vision, as postulated by the general hypothesis. In any case, stereo vision must have evolved because of the selective advantages it confers in particular ecologies. Below, we consider what these advantages may be.

## The functions of stereopsis

**Fig. 2.**
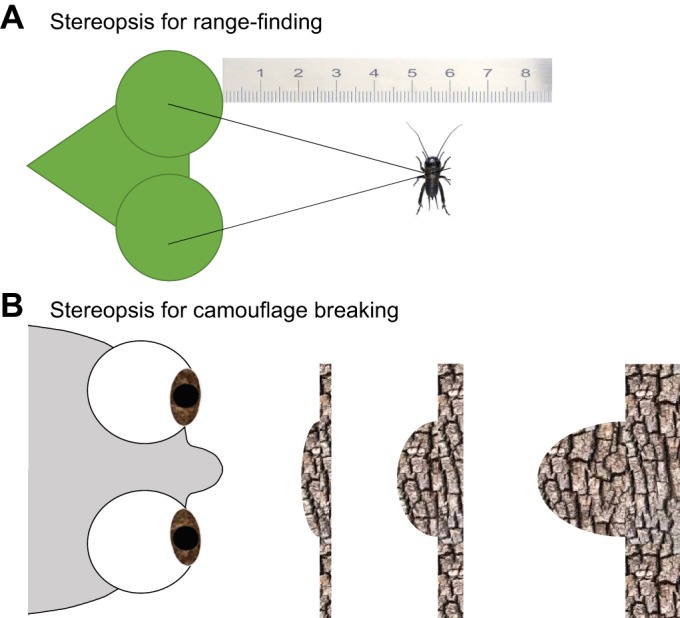
**Two fundamentally different functions stereopsis could theoretically subserve.** (A) Range-finding. Here, the intersection of lines of sight from the two eyes is used to derive the location of a viewed object in space; specifically, its metric distance from the observer. This requires knowledge of eye posture, and is thus more straightforward to implement in animals whose eyes are fixed in the head, such as the mantis shown. (B) Camouflage breaking. Here, stereopsis is used to derive the depth structure of a dense scene, say a beetle camouflaged against the bark of a tree. Calibration, including knowledge of eye posture, is required to obtain metric depth. Without this, the solution is ambiguous up to a relief transformation (Garding et al., 1995), so the distance of the tree and the shape of the beetle cannot be recovered (cf. the three example solutions shown). An uncalibrated stereo vision system can still detect the beetle as a bump on the surface, even though perfect camouflage would make it invisible monocularly.

The first species proven to have stereopsis were humans and other predators with front-facing eyes. This led some to hypothesise that stereopsis, and even binocular vision itself, evolved specifically to enable predators to detect prey (Cartmill, 1974). However, we now know that stereopsis has also evolved in lateral-eyed prey animals such as horses and sheep. In principle, stereo vision could perform several non-mutually exclusive functions (Fig. 2), which we discuss in more detail below.

### Range-finding

Most obviously, stereopsis could enable an organism to judge the distance to objects in its environment (Fig. 2A). In primates, for example, a suggested adaptive value that might have led to the evolution of stereo vision is that it enables prehension, the ability to judge distances and grasp objects, e.g. when moving between branches (Collins, 1921). More generally, distance measurement or ‘range finding’ is important in several other contexts, including navigation, prey capture and predator avoidance. Distance estimates from stereopsis could therefore be useful for many different animals.

Distance estimation by stereopsis is particularly straightforward for animals such as insects, whose eyes are fixed in place on the head and unable to rotate. In such animals, there is a fixed mapping from retinal disparity to distance. That is, once we know the positions of an object's two images on the retinae, we can immediately deduce its three-dimensional position relative to the animal's head, including how far away it is. For animals with mobile eyes, such as primates, the situation is more complicated because the mapping depends on the current eye posture (Fig. 1). Information about relative depth (e.g. which of two objects is closer) is available even if the eye position is unknown, but information about absolute depth or position in space requires an estimate of eye position, specifically, vergence (see Glossary). In principle, this information could be extracted either from extra-retinal signals such as proprioception from the eye muscles, or from a more complex analysis of the disparity pattern across the retina (Hartley and Zisserman, 2004; Read et al., 2009). Alternatively, a mobile-eyed animal could estimate an object's distance from the vergence required to fixate it (Fig. 1), without needing to measure disparity. Thus, vergence and disparity are distinct stereoscopic cues, analogous respectively to the accommodation and blur focus cues, discussed above. In practice, humans have a poor-quality estimate of vergence, and accordingly a poor ability to estimate metric distance solely from stereopsis (Bradshaw et al., 2000).

Toads and praying mantises both have fixed eyes, and so it is perhaps not surprising that, in both, stereopsis has been clearly implicated in judging distance for prey capture (Collett, 1977; Nityananda et al., 2016a; Rossel, 1983). Stereopsis might have been particularly selected for in these animals because both lie in wait for prey, which they try to capture if at the right depth. Toads do so with projectile extensions of their tongue, while mantises make a dynamic extension of their forelegs called a strike. For both of these animals, reliable depth information is fundamentally important to be able to judge the distance of prey before launching their predatory attacks to the right position. Frogs, and very likely toads, can also use knowledge of the elevation of a prey image on the retina to measure distance (Collett and Udin, 1988). For an object on flat terrain, the further the object, the higher it is imaged on the retina and, provided that the animal knows its own eye height, image elevation can be transformed into distance. As we have seen, triangulation-class depth cues are particularly reliable, but motion parallax would give away the predator's position and provide early warning to the prey. Because mantises have compound eyes, they do not have defocus-type cues. Toads are an interesting example as their simple eyes can move their lenses to accommodate and, in the absence of stereo cues, use this to gauge target distance. When present, however, stereo cues dominate over accommodation cues (Douglas et al., 1986; Jordam et al., 1980).

Contrast this with an animal such as the chameleon, which has exceptionally mobile, accommodating eyes. When a chameleon directs its eyes so that there is a binocular overlap, stereoscopic information is in principle available. However, because the eyes are highly mobile, there is no easy correspondence between the retinal position of the images in both eyes and the depth of an object. This would make computing depth in space from the retinal disparity extremely complex. Accordingly, chameleons have been shown to use accommodation cues (Harkness, 1977; Ott et al., 1998) and to lack stereopsis (Ott, 2001). Based on these arguments, we might therefore expect to find range-finding stereopsis in other ambush predators with fixed eyes that lack accommodation, like some species of spiders (e.g. crab spiders), and not in animals with accommodating, mobile eyes.

Combining stereoscopic distance information with the angular size of objects could allow animals to estimate absolute object size. This could be a strong selective force on animals that specialize on prey of particular size. Primates have such an ability (Tanaka and Fujita, 2015) – displaying a phenomenon called size constancy, where they can distinguish objects based on absolute size independent of the angle the objects subtend on the retina (McKee and Smallman, 1998). There is less evidence of this capability in other animals. Goldfish appear to be able to judge size even without binocular vision (Douglas et al., 1988) and toads seem to be able to judge the absolute size of gaps independent of the angle they subtend on the retina (Lock and Collett, 1980). In the praying mantis, by contrast, there appears to be no fixed preference based on a measurement of absolute prey size (Nityananda et al., 2016b). Instead there appears to be a response to smaller prey when nearby and larger prey when farther away.

### Camouflage breaking

**Fig. 3.**
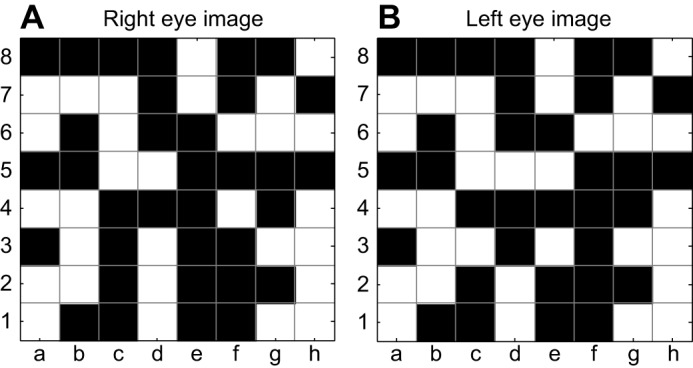
**Simplified example of a cyclopean stimulus.** The images are both ‘random chessboards’, near-identical apart from the region c3–f5. The 3×3 patch c3–e5 in the right eye's image (A) corresponds to the 3×3 patch d3–f5 in the left eye's image (B), i.e. this region has been shifted one square horizontally. Nothing identifies this patch as ‘special’ in either eye's image individually. If this image is viewed with the eyes crossed so that each eye views the image labelled for it, it is possible to see this patch floating in front of the page, although the percept is not compelling with the simple 8×8 chessboard used here for clarity.

As we have seen, stereopsis can provide a particularly precise and unambiguous estimate of distance (or at least relative depth, for animals with mobile eyes), but there are other depth cues that can often achieve the job just as well. This raises the question of whether there are other selection pressures favouring the evolution of stereopsis. In the 1960s, Bela Julesz revolutionised the study of stereopsis by drawing attention to its value in breaking camouflage. Julesz was prompted by his experience with aerial reconnaissance, where ‘the camouflaged target would jump out in vivid depth’ when viewed through a stereoscope (Babington-Smith, 1958; Julesz, 1971) (Fig. 2B). Using computers, Julesz created what he called ‘cyclopean’ stereograms (see Glossary), where a target is perfectly camouflaged in each eye individually, and is defined purely by the disparity between a region in left and right images. Fig. 3 shows a simple example (for clarity of exposition, this image consists only of 64 square elements; a much better depth percept is produced by similar images made up of hundreds of squares). Not only humans, but several other animals, including macaques, cats, horses, falcons and owls, perceive depth in such ideally camouflaged images (Clarke et al., 1976; Fox et al., 1977; Ptito et al., 1991; Timney and Keil, 1999; van der Willigen et al., 1998). Julesz suggested that camouflage breaking – as in revealing the beetle in Fig. 2B – is the reason that stereopsis evolved, rather than distance perception per se. Even in natural scenes where objects are not perfectly camouflaged, stereo vision can be a valuable aid to scene segmentation (Dal Mutto et al., 2011); by identifying sudden changes in depth, which often occur at object boundaries, stereopsis can help distinguish objects from their background and facilitate object recognition.

Julesz originally suggested that camouflage-breaking stereopsis ‘probably evolved in our insectivore primate predecessors (e.g. lemurs), rather late in the evolutionary timescale, in order to counteract the freeze response of insects’ (Julesz, 1995). The presence of camouflage-breaking stereopsis in herbivores such as horses obviously argues against this, and suggests that stereopsis may have evolved much earlier within mammals. Rather, it may be better to think more generally of stereopsis as aiding scene segmentation and providing 3D structure, with the ability to break camouflage arising as an extreme example of this. In primates and cats, this form of stereopsis is mediated by disparity-tuned neurons in primary visual cortex, which compute something close to the cross-correlation between local, filtered patches of the left and right retinal images (Cumming and DeAngelis, 2001; Qian and Zhu, 1997; Read, 2005). Similar matching metrics are also used in many machine vision ‘dense stereo’ algorithms (Scharstein and Szeliski, 2002). These correlation-based algorithms work well on most images, including natural scenes such as grass, where there is repetitive texture without particular objects or features. As Ives (1920) points out in a discussion of aerial reconnaissance, ‘small local elevations and depressions cannot be distinguished from mere difference in colour or marking. But with stereoscopic views these features [such as undulations of ground] stand out in a striking manner.’ One can imagine the selective advantages of this form of stereopsis to animals such as horses, which need to move at high speed over rough ground. The ability of horses to perceive perfectly camouflaged targets in cyclopean stereograms (Timney and Keil, 1999) may be a mere side effect.

## How many forms of stereopsis are there?

We have seen that stereopsis is found in a wide variety of species and appears to have evolved independently at least four times. Thus, it is entirely possible that stereopsis may have evolved differently in different taxa, or evolved divergently in different clades even where it originated in a common ancestor. Indeed, different forms of stereopsis may coexist within a given species. Certainly, human stereopsis seems to consist of a number of distinct modules using different stereoscopic cues, although the relationship between these is not yet entirely clear. In this section, we consider different forms of stereopsis.

### Is correspondence necessary?

**Fig. 4.**
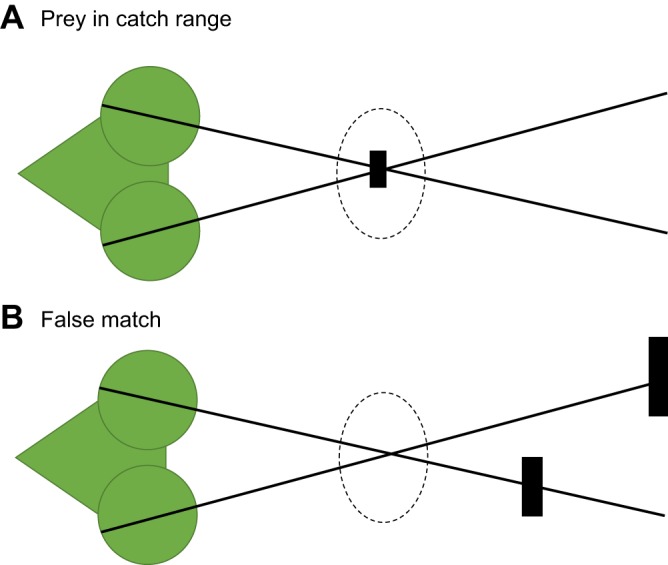
**False matches.** (A) A praying mantis views a prey item of its preferred size, located in the catch zone (dashed), at the fovea in both eyes. The heavy black lines indicate the lines of sight to the foveae. (B) The mantis views two larger objects much further away. There is nothing in the catch zone, but the foveal stimulus is the same. If the mantis simply struck whenever a prey item was detected in the fovea of each eye, it would strike in error to this stimulus.

The basic geometry underlying stereopsis is triangulation: following the lines of sight back from the two retinal images of an object to find where they intersect in space. This requires us to know which parts of the retinal image correspond to the same object in space. In complex natural scenes, solving this correspondence problem (see Glossary) is often challenging (Marr and Poggio, 1979; Scharstein and Szeliski, 2002). Could stereopsis evolve without correspondence? For example, praying mantises use their stereopsis to strike at prey when its image is within range of their spiked fore-limbs, at which point it falls at the fovea of each eye (Fig. 4A). One possible design for such behaviour would be to have a monocular ‘prey detector unit’, triggered by stimuli with the appropriate retinal size, luminance, speed, etc., centred on each fovea, and launch a strike when both are triggered together. This crude system does not solve correspondence, and so would fall victim to false matches (Fig. 4B). In fact, praying mantises do show evidence of solving the correspondence problem, so their stereopsis is more complex than this crude system (Collett, 1996; Rossel, 1996). Presumably, false matches are a serious enough problem in visual scenes as to produce a selection pressure favouring the evolution of stereo correspondence.

This example in the fixed-eyed mantis is related to the use of vergence in animals with mobile eyes. At first sight, vergence might appear to be a stereoscopic depth cue that does not require a solution to the correspondence problem; it simply requires an animal to know its own eye posture. But in order for this to be useful, the animal has to make sure that both eyes are fixating the same object, just as in Fig. 4. This requires at least basic correspondence.

One form of human stereopsis exploits a lack of correspondence. If you hold a finger in front of your face and close first one eye then the other, you notice that the finger occludes different parts of the background in the two eyes. Natural scenes typically contain many such occluded regions, which by definition have no corresponding match in the other eye. Humans can make qualitative depth judgements based on the position of these monocularly occluded regions (Harris and Wilcox, 2009; Nakayama and Shimojo, 1990; Tsirlin et al., 2012). This ability is known as da Vinci stereopsis, and we do not know whether it exists in other species. However, da Vinci stereopsis also depends fundamentally on correspondence: the occluded regions are only detected because the correspondence problem has been solved successfully over the majority of the image, where occlusions do not occur.

**Fig. 5.**
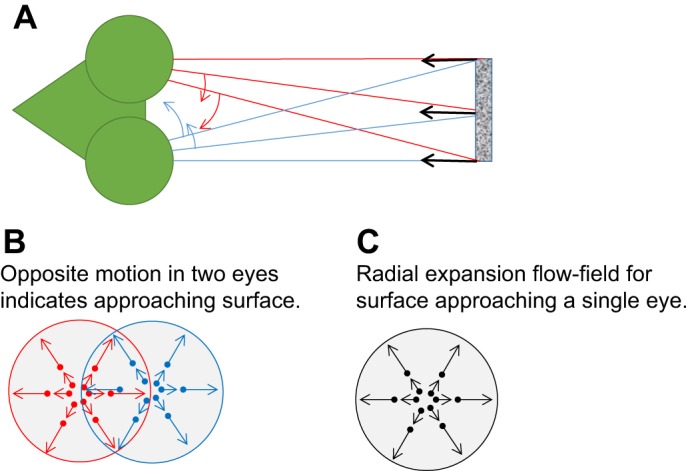
**Interocular velocity differences can indicate motion in depth without the need to compute disparity.** (A) As a theoretical example, we show a praying mantis, which has head-fixed foveae whose lines of sight are shown with the coloured lines. A surface directly approaching the mantis creates rightward motion on the medial (inner) surface of the left eye and leftward motion on the medial surface of the right eye, as indicated by the arrows (A,B). This is analogous to the radially expanding flow-field produced in a single eye approaching a surface (C), but here we are comparing the velocities of the same points in space viewed from different lines of sight. This makes the interocular velocity difference a stereoscopic depth cue.

However, a crude form of stereopsis is possible without any form of correspondence. An animal could discriminate whether it was approaching or receding from an object just by comparing the velocities in each eye (Harris et al., 2008). For example, leftward motion in the right eye and rightward motion in the left eye could indicate that the animal is approaching a surface head-on (Fig. 5A,B). This interocular velocity difference cue is closely related to the information available from a flow-field in a single eye (Fig. 5C), but is distinct because two different velocities are obtained for the same point in space. Primates appear to have a weak ability to detect motion in depth solely from this interocular velocity difference cue, although its independence from disparity is disputed (Harris et al., 2008; Shioiri et al., 2000; Czuba et al., 2014). Very little is known about whether other species use stereoscopic interocular velocity difference cues. Bees and flies do compare optic flow signals between their eyes (Srinivasan and Gregory, 1992; Hennig et al., 2011), but this is not a form of stereopsis, as it uses information from different regions of space, not the same region viewed from different angles. It is more closely related to the single-view flowfield shown in Fig. 5C, with the insect's lateral eyes effectively viewing different halves of the same visual sphere. In species with binocular overlap, the stereoscopic interocular velocity difference cue could potentially be an easy way to extract very basic information about the sign of stereomotion without solving the correspondence problem. However, information about the distance or speed of approach does require correspondence.

### How animals solve the stereo correspondence problem

As we have seen, in order to extract more than the most basic stereoscopic information, a stereo system has to work out which parts of the retinal image correspond to the same object. In primate stereopsis, correspondence begins in the primary visual cortex. Many neurons in this cortical area are sensitive to disparity, even in cyclopean images like more complex versions of Fig. 3 (Cumming and DeAngelis, 2001). These neurons have binocular receptive fields, i.e. they are sensitive to the retinal stimulus within a small patch of the left retina and a small patch of the right retina, and detect the correlation between the images in the two patches. When the images in the left and right receptive fields correspond to the same object in space, this correlation will be high. This is true whether the image is a real scene with many depth cues, or a highly artificial cyclopean stimulus. This explains why primate stereopsis is able to break camouflage (Cumming and DeAngelis, 2001; Parker, 2007; Tyler, 1991; Welchman, 2016). In fact, many features of primate stereopsis can be traced back to the properties of these neurons (Read, 2015a). For example, they have receptive fields at very similar locations in the two eyes, generally offset on the retina by less than half a degree. Human stereopsis can break camouflage only for similarly small disparities. Stimuli with a retinal disparity of more than approximately 0.5 deg appear double (Panum, 1858), i.e. we perceive both left and right images individually, rather than fusing them into a single whole. This is easily demonstrated by holding up a finger close to one's face while fixating on a distant object behind it; you will perceive two fingers.

### Contour versus cyclopean stereopsis

**Fig. 6.**
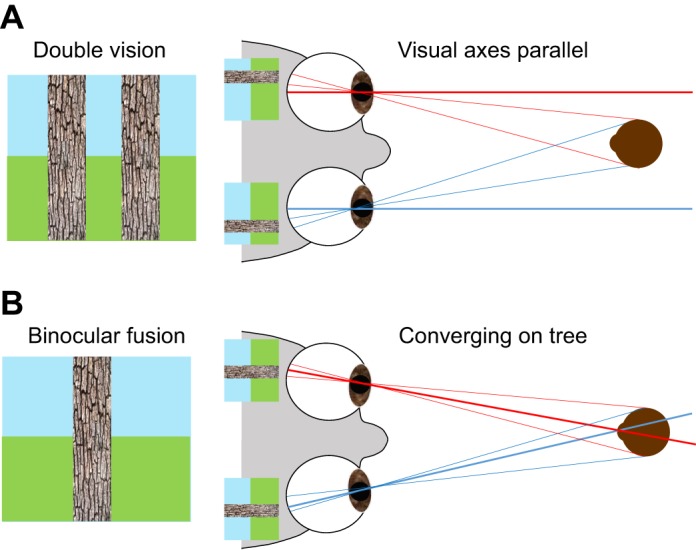
**Double vision.** Humans only experience single vision for a relatively small range of disparities. (A) Viewer fixates a distant point, so the visual axes (thicker lines) are parallel. Nearby objects, such as the tree shown, then appear double. Our coarse stereopsis can still use the monocular contours provided by the edges of the tree to recognise that the tree is nearby, but cannot extract the small disparity of, say, a beetle camouflaged against the bark. (B) Same scene after a convergence movement; now the viewer fixates the tree, bringing it within the operating range of fine stereopsis. Any lurking beetles can now be detected.

Intriguingly, however, human stereopsis does not fail altogether at large disparities. If a stimulus is briefly presented with a very large retinal disparity, up to 16 deg, it will appear double (Fig. 6A), but humans are still able to report the sign of its disparity – that is, whether it appeared nearer or further than the fixation point (see Glossary; Ogle, 1952a,b). This is only true for stimuli with relatively sparse, obvious monocular visible features, such as one or two thick lines marked on an empty background. This ability seems to be a completely independent form of stereopsis (Wilcox and Allison, 2009; Tyler, 1990; Read, 2015b). We shall refer to it as ‘contour stereopsis’ in order to discriminate it from the ‘cyclopean stereopsis’, which can break camouflage (although of course cyclopean stereopsis also works on stimuli with contours). Unlike cyclopean stereopsis, contour stereopsis does not appear to require a population of disparity-tuned neurons in primary visual cortex; it may be computed in sensorimotor and frontal cortices (Gamlin and Yoon, 2000). Intriguingly, it seems to operate in head-centric coordinates (Zhang et al., 2010). This means that retinal location is combined with estimated eye position in order to produce an estimate of head-centric direction, and depth is perceived based on this head-centric direction rather than the retinal disparity directly. Contour stereopsis may also be somewhat spared by disorders of binocular vision, such as strabismus and amblyopia, which are extremely disruptive to fine stereoacuity measured with cyclopean stimuli (Frisby et al., 1975; Giaschi, et al., 2013).

The purpose of this second, contour-based form of stereopsis may be to drive vergence. As we have seen, cyclopean stereopsis only works for a narrow range of disparities. Thus, before this fine, camouflage-breaking form of stereopsis can operate, our eyes must first fixate the object of interest. In the example shown in Fig. 6, the tree initially appears double (Fig. 6A), but its disparity can be detected by the contour stereo system, enabling the visual system to programme a convergence movement which brings it to the fovea in both eyes (Fig. 6B). Once the object is within fusional range, cyclopean stereopsis also contributes to vergence control. Cyclopean stereopsis can then also detect fine depth structure, such as the ridges and furrows of the bark, and even reveal the presence of a beetle that is perfectly camouflaged in each eye's view individually.

These two forms of human stereopsis are interestingly reminiscent of machine (computer) vision stereo algorithms. In ‘sparse’ machine vision stereo algorithms, distinctive features are identified in each eye's image individually, and are then matched up between eyes, potentially without any knowledge of how the two cameras are oriented with respect to one another, including their vergence. These matches can be used via a process known as ‘camera calibration’ to deduce relative camera pose (orientation and translation), which greatly constrains the set of possible disparities, reducing stereo correspondence from a 2D to a 1D problem. In a second stage, a ‘dense’ stereo algorithm can then extract disparity at every point in the image. Thus the vergence deduced by sparse stereopsis, as the orientation component of the camera pose, is used to reduce the range of disparities needed for dense stereopsis, much as the vergence triggered by contour stereopsis reduces the range of disparities needed for cyclopean stereopsis. (In animals, the translation component of camera calibration is a fixed interocular distance, so can be regarded as known.)

Barn owls also have cyclopean stereopsis, which, like primates', is based on identifying regions of left and right images that are locally highly correlated, and whose underlying neuronal mechanisms seem to be very similar. This is remarkable given that owl and human stereopsis evolved independently for predators with two very different anatomies and ecological niches. The obvious implication is that this form of stereopsis is optimal for animals with high acuity that are using stereopsis to extract 3D scene structure and/or detect camouflaged targets. We do not know whether owls also have a second, contour-based form of stereopsis. They may not need this because their ability to verge is extremely limited: no more than 4 deg, compared with up to 30 deg for humans. This gives them very limited scope to increase the range of their stereopsis by moving it around in space. Owl and primate cyclopean stereopsis both function over a range of disparities spanning approximately 1 deg (Nieder and Wagner, 2000). If owl eyes were completely fixed, these neurophysiology data imply that owl stereopsis would work from 40 to 170 cm and be optimised for a distance of 70 cm. Within this range, we would predict that owls should be better than primates at judging metric distances from purely stereoscopic information, because the mapping from retinal disparity to position in space would be fixed. In practice, owls' limited vergence may somewhat extend the range of distances over which their stereopsis is useful, with a corresponding decrease in the precision of metric distance estimates.

### Non-spatial correspondence

In principle, there are many ways to identify corresponding points in the images. For example, correspondences could be found based on matching luminance, contrast, texture, colour, motion or change of any property over time. Stereopsis as we know it from primates, cats and owls finds correspondences based on matching patterns of contrast over space, and is relatively insensitive to luminance. Yet might other systems match different aspects of the visual input?

Interestingly, no known biological stereo system appears to use colour to aid correspondence, even though on the face of it this could be used to help disambiguate false matches, and indeed is so used in some computer stereo algorithms (Bleyer et al., 2008). The only known invertebrate to possess stereopsis, the praying mantis, is also highly unusual among insects in that it appears to have only one class of photoreceptor and thus lack colour vision (Sontag, 1971; Towner and Gaertner, 1994). This raises the possibility that the neural machinery that subserves colour discrimination in other insects has been taken over to subserve stereopsis in mantises, perhaps because both processes involve difference computations (comparing the response in L versus M cones, or left versus right images) (Zhaoping, 2014).

Another interesting possibility is stereo correspondence based on motion. Humans can judge depth based on the disparity of a motion boundary in binocularly uncorrelated images (Halpern, 1991; Lee, 1970), an ability Lee referred to as ‘binocular-kinetic space perception’. Both of these can be viewed as examples of stereopsis based on disparity in the spatiotemporal rather than the purely spatial or contrast domain; ‘disparity’ here is the difference in position of a feature, such as a motion boundary, which does not correspond to an object in space. Very little is known about the neural basis of these forms of stereopsis, and – as in the discussion of interocular velocity difference above – it is not clear whether they reflect dedicated mechanisms which evolved to extract this form of information, or whether they are a side effect of purely spatial mechanisms. As a thought experiment, one can imagine training an artificial neural network to discriminate approaching/receding surfaces in dynamic random-dot patterns. Units within this network might well develop binocular space–time receptive fields that shift in opposite directions on the two retinae over time, in order to track changing disparity. If these units were then tested with uncorrelated stimuli, their shifting receptive fields would make them sensitive to depth defined by interocular velocity differences, even though these units had never previously been exposed to that cue and thus cannot have ‘evolved’ to extract it. In the same way, depth perception based on interocular timing differences (Falk and Williams, 1980; Morgan and Thompson, 1975; Morgan and Ward, 1980; Pulfrich, 1922; Read and Cumming, 2005) is thought to be a side effect of mechanisms that extract disparity in natural scenes. As in the discussion of horses and camouflage breaking, even if it can be demonstrated that an animal can exploit a particular cue, it can be difficult to determine whether this ability was actually selected for.

Lee (1970) argued that an animal whose visual system ‘is attuned to pick up the kinetic structure of the optic array directly’ might evolve purely ‘binocular-kinetic’ stereopsis. That is, it might be sensitive to the disparity of objects and boundaries defined by retinal motion, even if it had no stereopsis at all for images that were static on the retina. This is an interesting suggestion, especially in the context of non-human stereopsis. Human vision is relatively unusual in having high spatial resolution but fairly poor temporal resolution; we see detail best in static scenes, and the peak of our contrast sensitivity function corresponds to a relatively low speed (approximately 2 deg s^−1^; Barten, 1999), while our stereo vision has poorer resolution still (Kane et al., 2014; Norcia and Tyler, 1984). Accordingly, as Lee pointed out, research on stereopsis has concentrated on ‘time-frozen purely spatial’ optic arrays. Machine stereo algorithms also work almost exclusively on spatial information; for example, they are usually benchmarked by their performance on static pairs of images, rather than two streams of video information (Scharstein and Szeliski, 2002). Other animals have far better temporal resolution (e.g. approximately 170 Hz for dragonflies; Autrum and Gallwitz, 1951) and far lower spatial resolution. It might therefore make sense for them to base their stereopsis on temporal change, rather than the detailed pattern of contrast in the retinal images. However, at this point the existence of such a system remains speculative.

### Machine stereopsis

Machine stereo algorithms also provide examples of different forms of stereopsis (Lazaros et al., 2008). Modern computer stereovision algorithms already exceed the abilities of human stereopsis in many ways. For example, machine stereopsis can produce a high-resolution depth map across the visual field (Scharstein and Szeliski, 2002), whereas human stereopsis is limited to a narrow volume around the fixation point (Panum, 1858), deteriorates rapidly in the visual periphery (Blakemore, 1970) and has poor spatial resolution (Tyler, 1974). Machine stereopsis can be designed to work for arbitrary disparities and camera positions (Hartley and Zisserman, 2004), whereas human stereopsis is optimised for one particular eye posture and does not work at all for extreme eye positions (Phillipson and Read, 2010; Schreiber and Tweed, 2003). Machine stereopsis can benefit from chromatic information (Koschan et al., 1996), to which human stereopsis is largely insensitive (Lu and Fender, 1972).

However, human stereopsis outperforms machines in challenging situations such as detecting the disparity of a turtle on the river bed through a pattern of reflections on the water surface, or the disparity of a bird viewed through an interlacing pattern of leaves and branches at many different depths (Tsin et al., 2003). Thus, machine algorithms still have more to learn from human stereopsis. This process should be aided by the increasing level of detail at which computational neuroscientists are now able to describe the neuronal basis of primate stereopsis (Henriksen et al., 2016).

As we learn more about other species, it may prove that their stereopsis also has particular strengths that machine algorithms could learn from, reflecting the particular constraints and requirements of that species. For example, it seems likely that insect stereopsis is limited in its abilities but cheap in terms of computational resources, which might make it appropriate for low-power autonomous systems (Collett, 1996; Tippetts et al., 2016).

## Conclusions and future research

Several outstanding questions remain about stereo vision in animals. Studies have focused on a few species without a clear phylogenetic approach to see when and how many times stereo vision might have evolved. It is likely that there have been at least four independent evolutions of stereo vision. However, in order to assess just how widespread stereo vision is, we need more comparative studies with a greater diversity of animals (especially invertebrates). Studies of closely related species with different behavioural ecologies would be of particular interest. This would provide invaluable data about how many times stereo vision has evolved or been lost in response to different ecological selective pressures. It would also test how general the general hypothesis of stereo evolution actually is – are all animals that have binocular vision capable of stereopsis?

A related question is: what selective pressures lead to the evolution of stereopsis? Answering this would require studying the different animals that are capable of stereopsis and testing them for the different functions (e.g. range-finding, camouflage breaking) that have been hypothesised as selective pressures for its evolution. This would allow us to establish whether different lineages have evolved stereo vision for different functions, or whether there is a common selection pressure that led to its evolution in every lineage. As discussed above, one candidate for such a selection pressure is camouflage breaking. Thus far, we have evidence of this ability from almost every mammal and bird in which stereo vision has been demonstrated. Experiments investigating this in other animals such as toads and mantises would therefore be of fundamental importance towards testing camouflage breaking as a primary selective force for the evolution of stereopsis. Given that these animals require local image motion to find targets, which already breaks camouflage, it may be that camouflage breaking was not the driving force for their stereopsis, potentially meaning that they could have evolved a quite different form of stereopsis from our own.

**Fig. 7.**
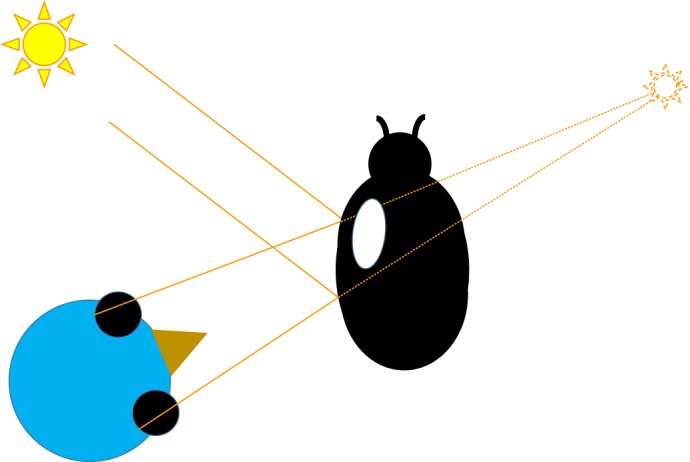
**Specular highlights on a convex surface appear behind the surface.** In this example, a bird views a shiny black beetle. The glossy highlights on the beetle's wingcase appear at different angles in the two eyes, indicating a bright source much further than the beetle. Potentially, this could cause a predator to misestimate the distance of prey.

The relationship between stereopsis and camouflage is also interesting in another way. The evolution of camouflage is a growing area of study (Skelhorn and Rowe, 2016), but we know next to nothing of how this has been influenced by stereo vision. Because stereopsis enables camouflage breaking in several species, it would therefore be a huge selective pressure in arms races between predators and prey. We should expect prey to evolve defences in response to such a selective pressure. What these might be and how widespread these defences are remain completely unknown. As noted, triangulation cues are hard to fool, but there are situations where they can mislead. For example, the virtual image of a light source on a shiny convex surface appears with a stereoscopic disparity indicating that it is more distant than the surface (Blake and Bülthoff, 1990). Thus, sunlight reflected off the glossy wingcase of a beetle might be perceived as a more distant object, potentially causing a predator to neglect it as out of range (Fig. 7). This particular suggestion is pure speculation, but the area could be a productive field for future research.

Finally, studying stereopsis in different animals should provide a window into the variety of mechanisms by which it is achieved. This would provide inspiration for new classes of machine stereo vision, which at the moment is almost entirely dominated by human-style stereopsis. As we have seen, both birds and mammals have evolved a form of ‘cyclopean’ stereopsis, which extracts spatial disparity based on the interocular cross-correlation of contrast information. Humans, and probably other mobile-eyed species, appear to have a second, ‘contour-based’ stereopsis system to aid in acquiring vergence. Other stereoscopic cues have been hypothesised, and some of these allow humans to perceive depth, albeit much more weakly. It remains to be seen whether any other animals have evolved distinctive forms of stereopsis primarily based on these or alternative mechanisms. In addition, it would be important to investigate how depth perception in different animals is aided by other non-stereoscopic cues and how depth processing is enabled by an interaction of stereo and non-stereo mechanisms in diverse animals.
